# An Alternating GluN1-2-1-2 Subunit Arrangement in Mature NMDA Receptors

**DOI:** 10.1371/journal.pone.0035134

**Published:** 2012-04-06

**Authors:** Morgane Riou, David Stroebel, J. Michael Edwardson, Pierre Paoletti

**Affiliations:** 1 Ecole Normale Supérieure, Institut de Biologie de l’ENS, IBENS, Paris, France; 2 Inserm, U1024, Paris, France; 3 CNRS, UMR 8197, Paris, France; 4 Department of Pharmacology, University of Cambridge, Cambridge, United Kingdom; Institute for Interdisciplinary Neuroscience, France

## Abstract

NMDA receptors (NMDARs) form glutamate-gated ion channels that play a critical role in CNS physiology and pathology. Together with AMPA and kainate receptors, NMDARs are known to operate as tetrameric complexes with four membrane-embedded subunits associating to form a single central ion-conducting pore. While AMPA and some kainate receptors can function as homomers, NMDARs are obligatory heteromers composed of homologous but distinct subunits, most usually of the GluN1 and GluN2 types. A fundamental structural feature of NMDARs, that of the subunit arrangement around the ion pore, is still controversial. Thus, in a typical NMDAR associating two GluN1 and two GluN2 subunits, there is evidence for both alternating 1/2/1/2 and non-alternating 1/1/2/2 arrangements. Here, using a combination of electrophysiological and cross-linking experiments, we provide evidence that functional GluN1/GluN2A receptors adopt the 1/2/1/2 arrangement in which like subunits are diagonal to one another. Moreover, based on the recent crystal structure of an AMPA receptor, we show that in the agonist-binding and pore regions, the GluN1 subunits occupy a “proximal” position, closer to the central axis of the channel pore than that of GluN2 subunits. Finally, results obtained with reducing agents that differ in their membrane permeability indicate that immature (intracellular) and functional (plasma-membrane inserted) pools of NMDARs can adopt different subunit arrangements, thus stressing the importance of discriminating between the two receptor pools in assembly studies. Elucidating the quaternary arrangement of NMDARs helps to define the interface between the subunits and to understand the mechanism and pharmacology of these key signaling receptors.

## Introduction

Ionotropic glutamate receptors (iGluRs) mediate most excitatory neurotransmission in the vertebrate brain and function by opening a transmembrane ion channel upon binding of glutamate. The iGluRs are critical for normal brain function and development, and their dysfunction is implicated in numerous neurological and psychiatric disorders [1]. Based on sequence homology and pharmacology, iGluRs can be grouped into three main subfamilies: AMPA-, kainate- and NMDA-type, the latter being unique in its ability to flux calcium and trigger synaptic plasticity mechanisms [1]. Since the cloning of iGluR subunits some twenty years ago, a wealth of information regarding iGluR structure and mechanism of operation has been obtained [2], [3]. Unlike other ionotropic neurotransmitter receptors that form either pentamers (Cys-loop receptors) or trimers (P2X receptors), the iGluRs assemble as tetrameric complexes composed of four homologous pore-forming subunits. All iGluR subunits share a unique modular architecture consisting of a large extracellular N-terminal domain (NTD) that participates in subtype-specific assembly and modulation; an agonist-binding domain (ABD also known as S1S2) that binds glutamate (or glycine/D-serine in certain NMDAR subunits); a transmembrane domain (TMD) comprising three membrane spanning segments (M1, M3 and M4) plus a short re-entrant loop (M2) lining the ion selectivity filter; and a cytoplasmic C-terminal domain (CTD), variable in length and involved in receptor trafficking, localization and regulation.

**Figure 1:**
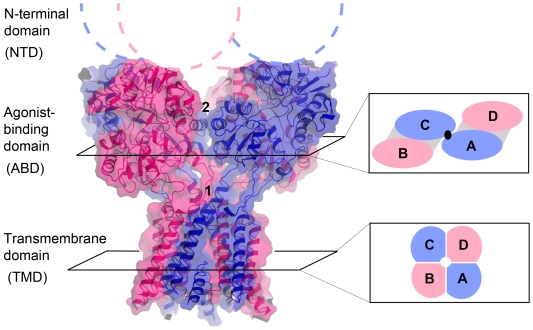
Subunit organization of the tetrameric GluA2 AMPA receptor. Side view of the gating core (ABD + TMD) of the GluA2 homotetramer [4]. The subunits adopt two different conformations, A/C (blue) and B/D (red). The insets on the right show how subunits are organized in the ABD and TMD (pore) layer. The grey shading indicates the two local ABD dimers. The black dot indicates the point of contact between subunits A and C at the dimer-of-dimers interface in the ABD layer. Numbers 1 and 2 highlight the two regions where mutations have been introduced in NMDARs.

A major breakthrough in the field was recently achieved with the crystal structure of a homomeric GluA2 AMPAR [4] thus providing the first atomic map of an intact iGluR. The structure revealed a massive Y-shaped structure in which the three major domains are arranged in layers: at the narrow “base”, the TMDs, at the “top” the NTDs and sandwiched in between these two layers the ABDs. As anticipated from studies on isolated domains [3], the extracellular NTD and ABD both assemble in the full length structure as dimers of dimers but with surprisingly few contacts between the four constitutive dimers. The pore region, in contrast, adopts a more compact structure with the typical four-fold symmetrical architecture found in potassium channels. Accompanying the two-fold to four-fold symmetry transition between the extracellular and pore regions, another key feature revealed by the GluA2 structure is domain swapping. Thus, local dimer assemblies in the NTD and ABD layers engage different subunit pairs. As a consequence of domain swapping and symmetry mismatch, the four chemically-identical GluA2 subunits adopt two pairs of conformationally distinct subunits, A/C and B/D (Fig. 1; [4]), with like conformers diagonal to one another. At the NTD layer, the A/C subunits are more “distal” from the central axis of the pore than the B/D subunits, while at the ABD layer, the A/C subunits occupy a more “proximal” position and participate in a tetrameric (dimer-of-dimers) interface (Fig. 1). Such an asymmetrical organization is without precedent in other families of neurotransmitter receptors.

NMDARs and kainate receptors are likely to adopt a general arrangement similar to that of AMPARs [1], [5]. However, unlike AMPA and kainate receptors that can function as homomers, NMDARs are obligatory heteromers usually composed of two GluN1 and two GluN2 subunits of which there are four isoforms (GluN2A-D) [6]. Although the NTDs and ABDs are likely to arrange as local GluN1/GluN2 heterodimers [7], [8], [9], [10], [11], there have been diverging results regarding the subunit order around the pore, with data supporting a 1/2/1/2 [4], [12], [13] or 1/1/2/2 [14], [15] organization. Here, by exploiting disulfide cross-linking to probe proximity relationships between subunits, we provide evidence that subunits in a functional GluN1/GluN2A receptor arrange in a 1/2/1/2 pattern with the GluN1 subunits adopting the “proximal” A/C conformation in the gating core region. Our results also highlight the fact that the existence of stable pools of intracellular GluN1 oligomers is a confounding factor in immunoblots studies of NMDARs.

## Results

**Figure 2:**
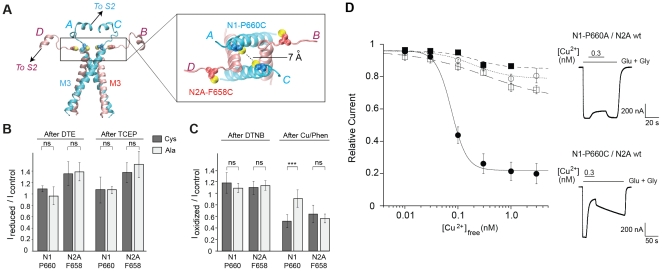
Insertion of a single cysteine in the GluN1 M3-S2 linker results in the creation of a high-affinity copper binding site. (**A**) The ion-channel (M3 and M3-S2 linkers) region of a modelled tetrameric GluN1/GluN2A receptor viewed from the side (left) and from above the membrane plane (right). In this alternating model (GluN1/2/1/2), the two GluN1 subunits (blue) are in the “proximal” A/C conformation and the two GluN2A subunits (red) in the “distal” B/D conformation. Homologous mutations GluN1-P660C and GluN2A-F658C are highlighted (sulphur in yellow). (**B** and **C**) Effects of reducing (**B**) and oxidizing (**C**) treatments on current amplitudes of the various cysteine (filled bars) or alanine (empty bars) mutant receptors. Values in **B** are (from left to right): 1.08 ± 0.05 (n = 8), 0.96 ± 0.15 (n = 7), 1.35 ± 0.2 (n = 14), 1.38 ± 0.16 (n = 8), 1.05 ± 0.22 (n = 9), 1.05 ± 0.06 (n = 11), 1.35 ± 0.16 (n = 20), 1.49 ± 0.2 (n = 6). Values in **C** are (from left to right): 1.18 ± 0.18 (n = 32), 1.09 ± 0.08 (n = 8), 1.1 ± 0.11 (n = 13), 1.13 ± 0.08 (n = 4), 0.51 ± 0.11 (n = 28), 0.90 ± 0.15 (n = 19), 0.65 ± 0.15 (n = 20) and 0.56 ± 0.08 (n = 8). (**D**) Copper inhibition dose-response curves for wild-type (open circles; Inhib_max_  =  21%, IC_50_  =  0.19 nM, n_H_  =  1.3), GluN1wt/GluN2A-F658C (open squares; Inhib_max_  =  34%, IC_50_  =  0.33 nM, n_H_  =  0.7), GluN1-P660C/GluN2Awt (filled circles; Inhib_max_  =  78%, IC_50_  =  0.075 nM, n_H_  =  3) and GluN1-P660A/GluN2Awt receptors (filled squares; Inhib_max_  =  15%, IC_50_  =  0.21 nM, n_H_  =  1.5). Right: current traces illustrating the inhibition of GluN1-P660A/GluN2Awt (top) and GluN1-P660C/GluN2Awt (bottom) receptors by 0.3 nM free copper. *** corresponds to *P*<0.001; ns, non-significant; Student’s *t*-test.

In the closed-state GluA2 structure, symmetry mismatch between the ABD and the TMD levels is mediated primarily by the short linkers connecting the two domains (S1-M1, M3-S2 and S2-M4 linkers; ref. [4]). In particular, the M3-S2 linkers adopt two strikingly different conformations with the linkers from the B/D subunits stretching away from the pore axis while the linkers from the A/C subunits remain close and prolong the TM3 segment by one helical turn (Fig. 2A). Accordingly, a cysteine introduced in the C-terminal end of TM3 (GluA2-M629C) can participate to a disulfide bridge in the A/C subunits but is too far removed to cross-link in the B/D subunits [4]. To explore which of the GluN1 and/or GluN2 subunits display the A/C and B/D conformers in the ABD-TMD linker region of a GluN1/GluN2A receptor, we introduced cysteines in GluN1 and GluN2A subunits at positions homologous to GluA2-M619 (Fig. 2A) and tested for possible disulfide cross-linking using electrophysiological recordings of *Xenopus* oocytes. Current responses from GluN1-P660C/Glu2Awt or GluN1wt/GluN2A-F658C receptors, and their respective control (alanine) mutants, were first subjected to the reducing agent dithioerythritol (DTE). Compared to control mutants, no significant effect of DTE on cysteine mutants was detected, and the use of Tris(2-carboxyethyl)phosphine (TCEP) as an alternative reducing agent confirmed this result (Fig. 2B). This lack of effect was surprising given that the GluN1-P660C mutation had previously shown to yield dimers on immunoblots [4], [13], and that cross-linking right next to the channel gate region (the SYTANLAAF region; ref. [16]) should lock the channels in a closed state.

Oxidizing treatments using 5,5'-dithiobis-(2-nitrobenzoate) (DTNB) to promote the formation of disulfide bonds also resulted in no significant effect on current amplitude. Intriguingly, however, the oxidizing complex Copper/Phenanthroline (Cu/Phen) selectively reduced currents carried by GluN1-P660C/GluN2Awt receptors but not by GluN1wt/GluN2A-F658C receptors (Fig. 2C). Because cysteines, through their sulfhydryl moiety, are commonly found in metal-binding sites [17], we wondered whether the effect of Cu/Phen on GluN1-P660C/GluN2Awt receptors could be accounted for direct copper inhibition rather than a redox effect. Incubating in copper alone produced similar inhibitions of GluN1-P660C/GluN2Awt currents than with Cu/Phen, and full dose-response curves with tricine-buffered copper solutions revealed the presence of a very high-affinity (subnanomolar) copper binding site in receptors containing the GluN1-P660C subunits (but not the GluN1-P660A subunits or GluN1wt) and the mutated GluN2A-F658C subunits (Fig. 2D). Thus, in functional receptors, the two cysteines introduced at position GluN1-P660 do not disulfide bridge as previously suggested [4], [13] but have their thiol groups in close enough vicinity to participate in a copper coordinating site. Altogether these results strongly favor a model in which, in the ABD-TMD linker region, the two GluN1 subunits occupy the proximal A/C positions while the GluN2 subunits occupy the more distal B/D position. Incidentally, we suggest that the reversion of the Cu/Phen effects on certain GluN1 cysteine pore mutants observed by Salussolia et al. [13] upon application of DTT may result from copper chelation rather than from a *bona fide* redox effect (see ref. [18] for the metal chelating properties of DTT).

**Figure 3:**
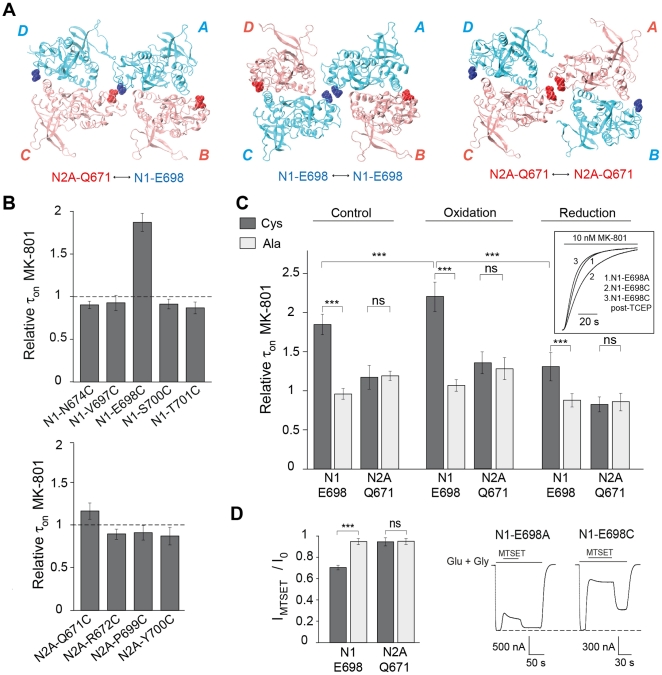
Disulfide cross-linking at the putative ABD dimer-of-dimers interface between GluN1, but not GluN2A, subunits. (**A**) Three possible arrangements viewed from the membrane region of GluN1/GluN2A ABD heterodimers in a NMDAR based on GluA2 structure. Mutated residues GluN1-E698 and GluN2A-Q671 are highlighted in blue and red, respectively. The residues either lie at the dimer-of-dimers interface in the proximal A/C subunits or protrude towards the exterior surface of the receptor in the distal B/D subunits. (**B**) MK-801 inhibition kinetics for various GluN1 (top) and GluN2A (bottom) mutants. Values are (from left to right): 0.9 ± 0.04 (n = 5), 0.93 ± 0.09 (n = 9), 1.85 ± 0.13 (n = 20), 0.91 ± 0.05 (n = 8), and 0.87 ± 0.06 (n = 7) for GluN1 mutants; 1.17 ± 0.15 (n = 21), 0.90 ± 0.06 (n = 11), 0.91 ± 0.09 (n = 11) and 0.87 ± 0.1 (n = 11) for GluN2A mutants. All GluN1 mutant subunits were co-injected with GluN2A wt and all GluN2A mutant subunits with GluN1 wt. (**C**) Effects of redox treatments on the MK-801 inhibition kinetics for cysteine (filled bars) or alanine (empty bars) mutants. Values are (from left to right): control conditions, 1.85 ± 0.13 (n =  20), 0.96 ± 0.07 (n =  12), 1.17 ± 0.15 (n =  21), 1.19 ± 0.06 (n =  9); after incubation with Cu/Phen (oxidation): 2.21 ± 0.18 (n =  12), 1.07 ± 0.07 (n =  10), 1.28 ± 0.14 (n =  18), 1.36 ± 0.14 (n =  9); after subsequent incubation with TCEP (reduction): 1.31 ± 0.18 (n =  19), 0.88 ± 0.08 (n =  7), 0.83 ± 0.09 (n =  17), 0.86 ± 0.11 (n =  9). Inset: MK-801 inhibition kinetics are slower in non-treated GluN1-E698C/GluN2Awt receptors (2) than in non-treated GluN1-E698A/GluN2Awt receptors (1) or TCEP-treated GluN1-E698C/GluN2Awt receptors (3) (normalized currents). (**D**) Effects of MTSET application on current amplitudes for cysteine (filled bars) or alanine (empty bars) mutants. Values are (from left to right): 0.70 ± 0.02 (n = 18), 0.95 ± 0.03 (n = 11), 0.95 ± 0.04 (n = 9) and 0.95 ± 0.02 (n = 5). Right: current traces illustrating the effects of MTSET on GluN1-E698A/GluN2Awt and GluN1-E698C/GluN2Awt receptors. *** corresponds to *P*<0.001; ns, non-significant; Student’s *t*-test.

We next introduced cysteines in the ABD layer to map contact points between neighboring subunits. Guided by the superposition of the GluN1/GluN2A ABD heterodimer onto the GluA2 structure (Fig. 3A), we focused on several residues in helices E and G of GluN1 and GluN2A subunits that could potentially participate in a dimer-of-dimers interface. At the level of this interdimer interface, three types of contacts can be envisioned according to the different possible arrangements of the two heterodimers in the tetrameric assembly: heterophilic GluN1-GluN2 contacts or homophilic GluN1-GluN1 or GluN2-GluN2 contacts (Fig 3A and see ref. [4]). To check for potential functional effects of cysteine introduction in the ABD region, we assessed the channel activity of mutated receptors by measuring the inhibition kinetics produced by the open-channel blocker MK-801 [19]. Of the several cysteine substitutions that were investigated, only GluN1-E698C/GluN2Awt mutant receptors showed a markedly altered (decreased) channel activity, as revealed by the 1.85-fold increase in MK-801 inhibition time constant compared with wild-type receptors (Fig. 3B). No such effect was observed with the control GluN1-E698A mutation (Fig. 3C). Moreover, channel activity could be further diminished (i.e. MK-801 inhibition time constant increased) by applying an oxidizing treatment, while a subsequent reducing treatment led to a strong increase in channel activity to a value close to that of untreated GluN1-E698A/GluN2Awt receptors (Fig. 3C). Thus, introduction of a single cysteine in the GluN1 subunit at position E698 is sufficient to result in disulfide bond formation. We also evaluated the effects of redox treatments on the activity of GluN1wt/GluN2A-Q671C receptors. In contrast to the effects previously described with the GluN1 mutant receptor, no effect specific to the GluN2A cysteine mutant was detected (Fig. 3C). These results indicate that within the ABD layer of functional GluN1/GluN2A receptors, residues from the GluN1 subunit ABD S2 region, but not the homologous region of the GluN2A subunit, participate in an interdimer interface. Thus, in a tetrameric GluN1/GluN2A assembly, the GluN1 subunits likely adopt the proximal A/C conformer at the ABD level, while the GluN2A subunits would adopt the more distal B/D configuration.

We obtained additional functional evidence that residue GluN1-E698 is involved in subunit-subunit contacts by reacting reduced (TCEP-treated) GluN1-E698C/GluN2Awt receptors with the thiol-specific modifying reagent MTSET. MTSET induced a marked and irreversible inhibition of currents from the cysteine mutant receptors, while the non-reactive alanine mutant receptors were barely affected. In contrast, no specific effect of MTSET was observed on GluN1wt/GluN2A-Q671C receptors (Fig. 3D).

**Figure 4:**
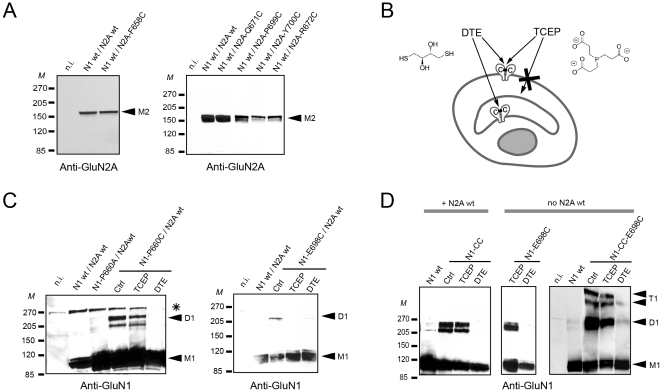
Intracellular and plasma-membrane pools of disulfide cross-linked receptors. (**A**) Immunoblots from *Xenopus* oocytes expressing either wt or GluN2A mutant receptors. (**B**) Schematic representation of a cell expressing intracellular and plasma-membrane pools of receptors with intersubunit disulfide cross-links. Because of their differential membrane permeability, the reducing agent DTE acts on both pools while the reducing agent TCEP acts solely on the cell surface pool. (**C**) Immunoblots from oocytes expressing either wt or GluN1 mutant receptors. (**D**) Immunoblots from occytes injected either with both GluN1 and GluN2A subunits or with the GluN1 subunit alone. M1 indicates the expected band position of the GluN1 monomer (∼110 kDa); M2, the GluN2A monomer (∼180 kDa); D1, the GluN1 homodimer (∼220 kDa) and T1 the approximate location of the GluN1 homo-trimer and/or homo-tetramer (>300 kDa). CC stands for mutations GluN1-N521C-L777C (in ABD intradimer interface). *M* stands for molecular weight marker and n.i. for non-injected oocytes. * indicates non-specific bands seen with the anti-GluN1 antibody.

We next turned to immunoblot experiments to directly assess cysteine disulfide cross-linking and intersubunit dimer formation. We first analyzed mutant receptors composed of wt GluN1 subunits and GluN2A subunits with a cysteine introduced either in the TM3-S2 linker region or the ABD region. Under non-reducing conditions, no formation of high-molecular weight bands was detected for any of the mutant receptors (Fig. 4A). This result indicates that the introduced cysteines in the GluN2A subunit do not participate in intersubunit disulfide bond formation, in good agreement with the results of the functional experiments using electrophysiology. In contrast, high-molecular weight bands were clearly detected with receptors harboring a single cysteine substitution either in the TM3-S2 or ABD region of the GluN1 subunit. These bands were attributed to disulfide cross-linked GluN1 homodimers since they ran at the expected size (∼220 kDa) and were absent in the control alanine mutants or in blots performed in reducing conditions (+DTE) (Fig. 4C).

The observation that GluN1-P660C mutant could form homodimers, while confirming previously published results [4], [13], was clearly at odds with our electrophysiological experiments, which indicate that in functional GluN1-P660C mutant the introduced cysteines form a metal binding site and not a disulfide bridge (see Fig. 2). Our 3D model also indicated that the two engineered cysteines are separated by too great a distance (7 Å S-S, 12 Å Cα-Cα; Fig. 2A) to cross-link [20], [21]. Because in electrophysiological experiments, only fully-mature plasma-membrane embedded receptors are monitored, we wondered whether the GluN1-P660C homodimers observed in immunoblots could be attributed to intracellular pools of cross-linked GluN1 subunits. It is well established that the GluN1 subunit, both in recombinant and native systems, can form stable intracellular homo-oligomers while GluN2 subunits do not [22], [23], [24], [25]. To try to discriminate between intracellular receptors from those expressed at the cell surface, we compared immunoblots from cells treated either with DTE or TCEP. Both compounds are powerful reducing agents but while DTE can cross biological membranes, TCEP is membrane-impermeant [26] (Fig. 4B). As shown in Figure 4C, the GluN1-P660C homodimer band was sensitive to DTE but not to TCEP, suggesting that the GluN1-P660C homodimers are for the most part localized intracellularly. The situation differed strikingly with the cysteine introduced in the GluN1 ABD at the putative interdimer interface (GluN1-E698C/GluN2Awt receptors). There, the GluN1-E698C homodimer band was sensitive to both DTE and TCEP (Fig. 4C). These results suggest that a sizeable fraction of GluN1-E698C/GluN2A receptors with disulfide cross-linked GluN1 subunits are expressed at the plasma membrane. These results are in good agreement with our previous observations that currents carried by GluN1-E698C/GluN2Awt receptors are redox sensitive (see Fig. 3).

We obtained additional evidence for the validity of our DTE/TCEP approach by expressing mutated GluN1 subunits alone, without a co-expressed GluN2 subunit. In this condition, the vast majority of the GluN1 subunits are known not to traffic to the plasma membrane but instead remain “stuck” in intracellular pools [22], [23], [24]. As anticipated, immunoblots of oocytes expressing the GluN1-E698C subunit alone revealed a GluN1 homodimer band sensitive to DTE but not to TCEP (Fig. 4D). When we combined the E698C mutation with two additional cysteine mutations within the ABD intradimer interface (mutations GluN1-N521C-L777C; refs. [7], [8], [27]), bands of very high molecular weight were observed in addition to the homodimer band. Again these bands were sensitive to DTE but not to TCEP (Fig. 4D). Thus, the triple cysteine GluN1 mutant subunit can accumulate as intracellular pools of homotrimers or homotetramers with disulfide cross-links within and between GluN1 ABD dimers. Noticeably, when the double mutant GluN1-N521C-L777C subunit was co-expressed with the wt GluN2A subunit, DTE-sensitive, but TCEP-insensitive, GluN1 homodimer bands were observed (Fig. 4D). These bands most likely correspond to intracellular stocks of GluN1 homodimers with the two GluN1 ABDs cross-linked at the intradimer interface. Such assemblies are not present in functional receptors. In these latter, the ABDs arrange as local GluN1/GluN2 heterodimers [7], [8].

## Discussion

In the present work we provide biochemical and functional evidence that mature GluN1/GluN2A NMDARs adopt an alternating GluN1/2/1/2 subunit arrangement with the two GluN1 subunits occupying the “proximal” A/C conformation seen in the AMPAR GluA2 receptor and the two GluN2A subunits occupying the distinct B/D conformation. Our biochemical approach based on differential detection of disulfide cross-linked receptors between intracellular and cell surface receptor pools also reveals that the subunit arrangement can differ between functional (cell surface) and intracellularly-retained receptors. In particular, cysteine-engineered GluN1 subunits have a strong propensity to form oligomeric assemblies that are not found in mature receptors. Overall, our results buttress previous findings that NMDAR maturation involves rearrangement of subunit contacts [25].

The interpretation of our results strongly relies on the assumption that the general subunit arrangement of NMDARs resembles that described in the AMPAR GluA2 structure, at least in the gating core region (ABD + TMD). The overall sequence homology between iGluR subunits and the striking similarities in the structure and mechanism of the ABDs between all iGluRs [1] strongly argue that this likely to be the case. Our present finding that, based on the GluA2 structure, cysteines (GluN1-E698C) introduced at a putative ABD tetrameric interface can cross-link in a mature GluN1/GluN2A receptor and impact its function (Fig. 3A) provides additional evidence that NMDAR and AMPAR subunits arrange similarly. It also suggests that in a NMDAR the ABD tetrameric interface experiences conformational mobility during receptor gating. Our results reveal that this interface involves the GluN1, but not the GluN2, subunits. Moreover, as a consequence of symmetry mismatch between the ABD and TMD layers, the GluN1 subunits - and more generally the A/C conformers in an iGluR - are expected to couple differently to the ion channel gate than the GluN2 subunits (B/D conformers). As a matter of fact, differential contributions of GluN1 and GluN2 subunits to NMDAR channel gating have been reported [28], [29], [30], [31]. The knowledge of the spatial organization of the subunits within a functional NMDAR complex provides an essential structural framework to understand how the four subunits interact and co-operate during channel gating and modulation.

## Materials and Methods

### Molecular biology

The expression plasmids for the rat GluN1-1a subunit and the rat GluN2A subunit, the mutagenesis strategy and sequencing have been previously described [10], [18]. All amino acid (aa) numbering is for full-length proteins (i.e. including the signal peptide). For correspondence with other studies not considering full-length proteins, a difference in the numbering of GluN1 and GluN2A subunits should be applied (for ref. [13], +18 and +19 aa, respectively; for ref. [4], -1 and +27 aa, respectively).

### Electrophysiological experiments and data analysis

Recombinant NMDARs were expressed in *Xenopus* laevis oocytes after nuclear co-injection of cDNAs (at 10-30 ng/µl) coding for the various GluN1-1a and GluN2A subunits (ratio 1∶1). Oocytes were prepared, injected, voltage-clamped, and superfused as described previously [18]. The standard external solution contains (in mM): 100 NaCl, 0.3 BaCl_2_, 2.5 KCl, 5 HEPES, and 0.01 DTPA (pH 7.3). The heavy metal chelator DTPA (diethylenetriamine-pentaacetic acid) was added to all solutions, except in copper-containing solutions, to prevent tonic inhibition of GluN1/GluN2A receptors by trace amounts of zinc [18]. NMDAR-mediated currents were induced by simultaneous application of saturating concentrations of L-glutamate and glycine (100 µM each). Recordings were performed at a holding potential of −60 mV and at room temperature. For copper dose-response experiments, copper was buffered using 10 mM tricine. For free copper concentrations of 0.01, 0.03, 0.1, 0.3, 1 and 3 nM, total (added) concentrations of copper (CuSO_4_ salt) were (in µM): 2.3, 7.0, 23, 70, 210 and 550, respectively. These values were calculated using Geochem with stability constants of 10^–7^ M and 10^–4.2^ M for the equilibria Tricine + Cu^2+^ ? Tricine-Cu^2+^ and Tricine-Cu^2+^ + Tricine ? Tricine_2_-Cu^2+^, respectively [32]. For the highest concentrations of free copper (1 and 3 nM), pH was readjusted to 7.3. Experiments using MK-801 (10 nM) and ([2-(Trimethylammonium)ethyl]methane-thiosulfonate bromide) (MTSET, 200 µM) were performed as described in ref. [10].

Data were collected and analyzed using Clampex 7.0 and Clampfit 9.2 (Axon Instruments), respectively. For copper dose-response curves, data points were fitted in Kaleidagraph 4.0 (Synergy Software) using the following Hill equation: I_Cu_/I_control_  =  0.96-Inhib_max_/(1+(IC_50_/[Cu^2+^]^nH^), where I_Cu_/I_control_ is the mean relative current, [Cu^2+^] the concentration of free copper, n_H_ the Hill coefficient and (Inhib_max_ + 0.04) the maximal copper inhibition. IC_50_, Inhib_max_ and n_H_ were set as free parameters. Error bars represent the standard deviation of the mean value.

### Redox treatments

Oocytes were incubated at room temperature with either dithioerythritol (DTE, 5 mM, 15 min), tris(2-carboxyethyl)phosphine (TCEP, 5 mM, 15 min), 5,5'-dithio-bis-(2-nitro-benzoic acid (DTNB, 0.5 mM, 5 min), or copper(II):phenanthroline (Cu/Phen, 10∶30 µM, 12 min), all prepared in a Barth solution supplemented with gentamycin (50 µg/ml) and D-AP5 (50 µM) [8]. For DTE and TCEP solutions, pH was adjusted to 8.0. The DTNB solution was also supplemented with DTPA (10 µM).

### Immunoblotting

Sample preparation, non-reducing SDS-PAGE, and immunoblotting were performed as described in ref. [10]. The following antibodies were used: anti-GluN1 antibody (1∶1000, mouse monoclonal MAB363 clone 54.1; Millipore), anti-GluN2A antibody (1∶500, rabbit monoclonal A12W; Millipore), secondary goat peroxidase-conjugated anti-mouse or anti-rabbit antibody (1∶20,000, Jackson ImmunoResearch). Non-specific bands (i.e. bands detected in immunoblot experiments with non-injected oocytes), were regularly observed with the anti-GluN1 antibody. The intensity of these bands varies from one batche of oocytes to another. The molecular weight of the main non-specific band is ∼270 kDa.

### Homology modeling

3D homology modeling was performed using the program Modeller and structure illustrations were prepared using PyMol and VMD (see ref. [33]). The model of the NMDAR pore region (Fig. 2A) was built by homology to the GluA2 structure (pdb 3KG2; ref. [4]) while the three different arrangements of ABD tetramers (Fig. 3A) were built by superimposing two GluN1-GluN2A ABD heterodimers (pdb 2A5T; ref. [7]) on the GluA2 ABD tetramer.
